# Considerations when Measuring Biocatalyst Performance

**DOI:** 10.3390/molecules24193573

**Published:** 2019-10-03

**Authors:** Mafalda Dias Gomes, John M. Woodley

**Affiliations:** Department of Chemical and Biochemical Engineering, Technical University of Denmark (DTU), DK-2800 Kgs Lyngby, Denmark; macoad@kt.dtu.dk

**Keywords:** biocatalysis, immobilized enzymes, turnover number, process performance metrics, enzyme kinetics, enzyme stability

## Abstract

As biocatalysis matures, it becomes increasingly important to establish methods with which to measure biocatalyst performance. Such measurements are important to assess immobilization strategies, different operating modes, and reactor configurations, aside from comparing protein engineered variants and benchmarking against economic targets. While conventional measurement techniques focus on a single performance metric (such as the total turnover number), here, it is argued that three metrics (achievable product concentration, productivity, and enzyme stability) are required for an accurate assessment of scalability.

## 1. Introduction

In recent years, the field of biocatalysis, which uses one or more enzymes to convert substrates to higher value chemical products, has become established as an alternative to homogeneous (or heterogeneous) catalysis in organic synthesis. The driver behind this exciting development has been both the demand for more selective chemistry (especially in the pharmaceutical sector), as well as for greener chemical processes, encompassing the principles of sustainability [1,2]. Most recently, the development of protein engineering has made it possible to modify enzymes, for example, by changing some of the specific amino acids (of the many that make up the molecular structure) or by shuffling variants. Modification can follow a path of directed evolution which accelerates the selection of improved enzymes against a given trait or property. Such a strategy has been focused on screening for enzymes with the ability to convert non-natural substrates into useful products, and even carry out non-natural reactions [3], although specific traits can also be evolved, dependent upon the starting point [4]. Despite the enormous progress that has been made, many reactions remain in the laboratory, due to the difficulties of translational research. Unsurprisingly therefore, industrial applications thus far have been mostly focused on higher-priced products, such as pharmaceuticals [5]. Nevertheless, as the field develops, an increasing number of medium-priced products are also being produced using biocatalysis [6]. One of the challenges of translating an interesting laboratory reaction into an industrial process is a characteristic feature of enzymes, namely their limited stability outside a narrow operating window with respect to temperature, solvents, pH, ionic strength, and salt type [7]. Indeed, the operational stability of an enzyme is one of the most important parameters by which to assess the potential of a given biocatalyst for industrial applications [8]. In general, the lower the value of the product, the more important it is to use a stable enzyme [9]. While operational stability can be improved by immobilization and also by protein engineering [10], measuring stability and understanding its impact on the final process is more complicated. This is particularly the case for immobilized enzymes, where the method of immobilization and the support material may affect the observed enzyme properties.

Although catalytic reactions have frequently been proposed as a means to achieve green synthesis, all catalysts in industrial processes represent a certain contribution to the operating costs. In other words, for every kg of product made, the catalyst represents a certain percentage of the cost. How much they contribute depends on the cost of the catalyst (usually per mass of commercial material) and the effectiveness with which it is used (i.e., how much product is made by the catalyst). Nevertheless, for lower-priced products (such as bulk chemicals or fuels), this may prove critical to implementation. An interesting example was reported in an excellent paper estimating the cost contribution of an enzyme to the product in the production of lignocellulosic biofuels [11]. Here, it is clear that unless the cost contribution of the enzyme is low enough, its implementation is not possible. Hence, understanding the performance of a biocatalyst in a given case is often of great importance to its ultimate implementation on an industrial scale. In general, for enzymes, two metrics are frequently used to describe the performance. The first concerns the rate of the catalyzed reaction (compared to the uncatalyzed one), and is often stated as the ‘activity’ of the enzyme. Activity is quoted as the rate of the reaction when converting a given standard (natural) substrate to a product. The substrate chosen is dependent upon the particular enzyme and the type of reaction. The second metric commonly used concerns the ‘stability’ of the enzyme, and is often stated as the thermodynamic (or conformational) stability (most often expressed as the melting temperature of the protein). The melting temperature represents the transition between the native and unfolded states of the protein. However, while these two metrics are interesting for enzymologists, they turn out to be less useful for the application of enzymes in chemical processes, i.e., in biocatalysis. First, the conditions under which biocatalytic reactions occur in industry are usually far from those in nature; this needs to be taken into account. Secondly, although it is a necessary condition to run a reaction under the melting temperature, it is not sufficient, and enzyme stability requires a metric which is closer to the process and operational conditions, which of course may vary from reaction to reaction. This is termed operational stability, and it is particularly important that it is measured under relevant conditions in order to be useful. Finally, it is already clear that immobilized enzymes in particular require a deeper level of analysis.

The original rationale for immobilization was based on the need for simplifying the separation of soluble enzymes from product streams in order to facilitate recycling. In many cases, a side benefit was improved stability. Today, the arguments are a little different. First, it is clear that immobilization remains of interest in the implementation of pharmaceutical processes. Although the cost of enzymes may not be so critical in this sector, there remain other reasons why immobilization is of interest. For example, today it is of particular importance to contain enzyme(s) within the plug-flow tubes in which flow biocatalysis is carried out [12,13,14]. Such an approach builds on the many benefits of flow chemistry, which is already widely implemented in other pharmaceutical process development programs [15,16]. Immobilizing the enzyme in this way also ensures the effective removal of any protein prior to the next operation. Likewise, cascades of enzymes which are increasingly used in synthesis benefit from the containment of individual enzymes in order to avoid cross reactions. Secondly, as biocatalysis becomes increasingly effective, driven by the latest tools in protein engineering, it becomes possible to use enzymes for the production of lower-priced products. For these products, immobilization can also be highly beneficial, since the cost contribution of the enzyme(s) will prove to be significant. These two factors now mean that there is a revival of interest in enzyme immobilization. In such systems, it has long been known that diffusional limitations can exist (reducing the observed activity), while stability in many cases is improved. Diffusional limitations with immobilized enzymes can also occur when converting polymeric substrates [17]. Although such trade-offs do not exist in all cases, especially at high protein loads [18], they emphasize the need not only to examine the total amount of product that can be made using an enzyme, but also, the activity, as well as the stability, since both are important. For immobilization, other considerations are also necessary, such as potential mechanical fragility, moderate hydrophilicity, and, critically in some cases, dissolution in organic media.

In this brief paper, it will be argued that a change is required in the way we describe biocatalyst performance, so as to provide useful data for further biocatalyst and process improvements. Such an improvement strategy will prove vital for the further implementation of new biocatalytic processes in order to facilitate the translation from laboratory synthesis to industrial scale production process.

## 2. Motivation for the Assessment of Biocatalyst Performance

When considering biocatalysis alongside homogeneous (or heterogeneous) catalysis, although some overlap in operating conditions (such as pH, temperature and pressure) is possible between reactions [19], in general, each type of catalysis operates in its own operational niche (e.g., biocatalysis operates at moderate temperatures, while heterogeneous catalysis frequently requires higher temperatures). For this reason, few direct comparisons between the performance of homogeneous (or heterogeneous) catalysts and biocatalysts have been undertaken. Nevertheless, as the field of biocatalysis moves to lower-priced products, it is likely to become increasingly important to do so. Based on the established principles of retrosynthesis [20], but now also involving enzymes, new synthetic pathways are becoming increasingly possible using multiple enzyme steps [21,22,23]. In some cases, pathways will best be expressed in microbial hosts, while in other cases, better as a cascade of multiple enzymes. New routes will be of great importance in the creation of sustainable pathways from renewable feedstocks and building blocks to valuable products. Evaluating a given route to a molecule will be determined not only by an assessment of thermodynamic limitations and operating conditions, but also by evaluating catalyst performance. Hence, it could be useful to establish a basis for direct comparison. A second motivation to establish a good way to measure the performance of a biocatalyst is that we increasingly need to compare different biocatalysts with each other. In some cases, these might involve different enzymes classes. For example, the production of (chiral) amines (of great importance to industry) can be carried out using many different types of biocatalysts (e.g., transaminases, amine dehydrogenases, imine reductases, and amine oxidases), and it would be valuable to be able to compare these directly when working on the same synthesis to assist route selection [24,25,26]. In other cases, enzyme variants made by protein engineering will need to be compared. The final motivation to measure biocatalyst performance is to be able to benchmark performance compared to other known reactions or against economic targets for a given process. This can provide the basis for further improvements of enzyme performance via protein engineering, and is therefore, of great importance in the development of new biocatalytic processes [27].

## 3. Existing Methods for Measuring Biocatalyst Performance

Several options currently exist for measuring biocatalyst performance in a given process, although many reports focus on a single metric in a given case. Biochemists have long used so-called catalytic efficiency (also termed specificity constant, expressed as k_cat_/K_M_, from the terms in the well-known Michaelis-Menten rate law). Of course, this may be misleading, since it says nothing about the substrate concentration range over which this is measured. In general, in an industrial process, it is necessary to minimize downstream product recovery costs, and consequently, it is essential that products are produced at high enough concentrations; therefore, a sufficient concentration of substrate should be used. This is usually greatly in excess of K_M_, meaning that dividing k_cat_ by K_M_ is no longer relevant [8]. However, there are also some exceptions in which knowledge of K_M_ specifically can be important:

1. When converting non-natural substrates, it might be of great importance, because the K_M_ might be particularly high (low affinity).
2. When poorly water-soluble substrates are supplied from a second organic phase, it is important to know K_M_ in order to ensure that the enzyme is used effectively.
3. When enzymes are used for degrading specific compounds in waste streams, the final required concentration of the substrate in the effluent stream may be well below K_M_, resulting in an inefficient use of the enzyme.
4. When high conversions of substrate to product are required, the final part of the reaction may be carried out with substrate concentrations below K_M_, affecting the amount of enzyme required.
5. When using an enzyme in bimolecular reactions, the K_M_ on both substrates needs to be checked to ensure that the concentration of one of the substrates is not beneath its K_M_.

In fact the K_M_ can range from µM to mM values, dependent on the enzyme used and substrate being converted. In any case, k_cat_/K_M_ does not indicate how long the enzyme can be used. In order to understand this, it is necessary to examine the change in k_cat_ over time, referred to as the enzyme operational stability, as discussed previously. Whether operational or thermodynamic stability is measured, in both cases, it is well established that operating conditions, as well as modifications to the enzyme (either through protein engineering or immobilization), can greatly affect the stability [28].

In other types of catalysis (e.g., heterogeneous catalysis), the emphasis has been placed more on the number of moles of substrate that can be converted per mole of catalyst (so-called turnover or TON). Although frequently quoted also in biocatalysis, it raises a number of questions. For example, what is the dependence of the TON on operating conditions; furthermore, it says nothing about the speed of the reaction, just the amount of product that can be produced from a certain amount of catalyst. Hence, quoting TON and the rate together could be more useful. This is more commonly combined into a single metric, termed turnover frequency (TOF), and can be defined at TON/time (for an enzyme catalyst, it can also be expressed at the Michaelis-Menten rate constant, k_cat_). If the catalyst is stable, then TOF will be a valuable measure. However, typically, this is not the case, and so a more useful term is the total turnover number TTN, which can be defined as the total moles of product produced per mole of enzyme over the entire lifetime of the enzyme. The total turnover number allows for catalyst degradation over time, and therefore, when using enzymes, this is particularly helpful. An excellent discussion of the role of TTN in biocatalysis is given in the seminal review by Bommarius [8]. It has also been suggested that using data on kinetics combined with independent data on operational stability enables the prediction of TTN [29]. In different reports in the scientific literature, all three metrics (TON, TOF and TTN) have been used variously to define enzyme performance. Interestingly, a recent paper compared these three metrics for the enzymatic conversion of 2,5-furandicarboxylic acid from 5-methoxymethylfufural using a self-sustained enzymatic cascade [30]. The paper clearly indicates that while in some cases TOF is high, the corresponding TTN may also be high, even with a poor stability (expressed as a low value of half-life). The reverse is also shown, i.e., that with an enzyme with a low TOF, despite a higher stability, the TTN remains low. This emphasizes that TTN alone is a poor measure of catalyst performance. Indeed, both activity and stability contribute to the final numerical value of the term. Furthermore, TTN is dependent upon the operating conditions. Interestingly, a recent paper in the catalysis field highlighted the need for a standardized way of measuring TON and TOF [31], and there is no reason this could not be extended also to TTN. The standardization of definitions in this way could be of great value in the long run.

## 4. Use of Biocatalyst Yield

One of the virtues of using TTN to describe biocatalyst performance is that it can be used to guide further protein engineering, as suggested by Rogers and Bommarius [29]. Nevertheless, establishing the need for, and extent of, protein engineering requires a correlation between TTN and the cost of the enzyme. Herein is a limitation with the TTN method, i.e., that everything is calculated per mole of enzyme (or in some cases, per active site). However, enzymes are sold on the basis of mass (or sometimes activity), and therefore, not only is the molecular weight of the enzyme required, but also knowledge of its purity. For commercial enzymes, this is rarely the case, since impure enzymes are usually used in the process as ‘crude’ cell lysate (so as to minimize costs). One solution is to redefine TTN on a mass basis (i.e., the mass of product produced per mass of enzyme preparation used over its lifetime). Bommarius has termed this the ‘productivity number‘ (PN) [32], but it is perhaps more instructive to see it as a kind of yield (product produced/enzyme consumed over the lifetime of the enzyme), and in several recent papers, we have termed this ‘biocatalyst yield’ (BY) [9]. Other terms could also be used, such as the biocatalyst (lifetime) output. This is of particular relevance because the application of enzymes (biocatalysis), rather than the study of enzymes (enzymology) uses cell lysates, necessitating an alternative metric. However termed, the ‘biocatalyst yield’ (or its equivalent) has a direct relationship to the cost of the enzyme and is, therefore, a very practical way to measure the performance of an enzyme for a process chemist or engineer. Target values depend now not only on the price of product being produced, but also on the cost of the enzyme (itself being dependent upon format and purity). For enzymes used in the whole-cell format, or those which are immobilized, this may indeed prove to be the only way of making useful benchmarks and comparisons. Table 1 lists the alternative methods for measuring biocatalyst performance. Still, when making such measurements, decisions need to be taken regarding what constitutes the useful lifetime of the biocatalyst. Using such measures is a first step towards the evaluation of biocatalyst performance, but it turns out still not to be sufficient.

## 5. Complementary Metrics

### 5.1. Product Concentration

Of all the measurable indices of the potential commercial success of a chemical reaction when transferring from laboratory to pilot or industrial scale, one of the most important is the product concentration. This is particularly the case for biocatalytic processes, where product recovery is frequently from aqueous solutions. Many reports on new biocatalysts now emphasize the importance of this [33,34,35]. In itself, the target product concentration required will determine the substrate concentration that is needed, and thereby, the conditions of the reaction under which a biocatalyst should be tested to determine the biocatalyst yield. Nevertheless, in many cases, substrate inhibition is observed at high concentrations, meaning substrate feeding may be required or else supplied via a second liquid phase, complicating the measurements. Even more problematic is the fact that products may prove inhibitory, and therefore, in the first instance, the reaction needs to be stopped, the product removed, and the reaction run again with the same enzyme. Repeat operations in this way of course increase the biocatalyst yield. Hence, to determine the biocatalyst yield, it is also necessary to know the achievable product concentration. Alternative strategies could involve in situ product removal (where the inhibitory product is removed and separated while the reaction is in progress [36,37]), which, if implemented, need to be incorporated into the measurement strategy.

### 5.2. Productivity

**Figure 1:**
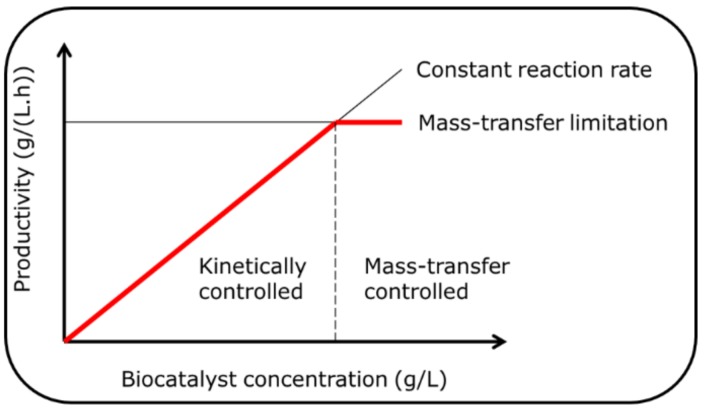
Productivity as a function of biocatalyst concentration. The red line indicates the observed productivity of the biocatalyst. The vertical dashed line indicates the boundary between the kinetically-controlled (left-hand side) and the mass–transfer controlled (right-hand side) of the plot.

Even if the product concentration is known, and it is therefore known how many times the enzyme must be recycled to achieve a given biocatalyst yield, this is still not adequate for benchmarking the process in economic terms. This is because the biocatalyst yield in itself says nothing about the time the reaction takes. As a true catalyst, if the enzyme concentration is doubled, then the rate of product formation will double. Productivity (defined as the amount of product produced per volume of reactor per time) is therefore also doubled. Hence, to achieve sufficient productivity in a process, the amount of enzyme used can simply be increased. This has a direct effect upon the biocatalyst yield. However, provided that the relationship between productivity and the mass of the biocatalyst is linear, the biocatalyst yield will be maintained. In some cases, it may even be that operational stability is affected by the enzyme concentration. Nevertheless, with respect to productivity, there are two occasions when particular care should be taken:

1. The first is when the reaction rate is sufficient to become limited not by the enzyme reaction rate, but by the mass transfer of the substrate to the enzyme. This is schematically shown in Figure 1. This is the case with many immobilized enzymes where diffusional limitations mean that the enzyme may operate at less than the maximum rate of reaction. Hence, in such cases, it is always good to evaluate the enzyme activity both with a soluble and an immobilized enzyme. Interestingly, improvements in enzyme activity due to protein engineering make this problem more complicated, since for the same load of protein, the maximum possible activity will be higher. Likewise, with poorly-water soluble substrates, problems can arise. The well-known case of oxygen supply to oxidases (or oxygenases) highlights this point well. Here, the maximum transfer rate of oxygen is limited by the low water solubility of oxygen, meaning that the maximum rates will always be low (even in cases with a high mass transfer coefficient of oxygen from a gas to aqueous phase). A more detailed explanation is given in several recent publications on the topic [38,39].
2. The second case is when the amount of protein loaded in the reactor exceeds what is practical from an operational perspective. For example, an immobilized enzyme will reach a maximum in a stirred tank at around 10% by volume. Above this value, particle-particle collisions will result in attrition (making downstream filtration problematic). Likewise, in a packed bed, to allow for the void space between particles and to achieve adequate flow through the bed, a maximum of around 60% by volume is to be expected. A soluble enzyme can also have an upper limit, dependent upon downstream recovery (ultrafiltration) or removal strategies. In pharmaceutical processes, regulatory demands mean that all residual protein must be removed from solution prior to the final processing steps and product formulation.

Target values for productivity are determined by the amount of product required in a given time, as well as the size of the reactors to be used. The size of the reactors is a clear indication of the capital costs (or else toll manufacturing costs) in a given case. Target productivities depend very much on the value of the product being produced (itself being linked to the mass of the product required).

## 6. Towards a Systematic Approach

### 6.1. Initial Tests of Intrinsic Metrics

It is clear that initial assessment of an enzyme performance in the laboratory should focus on the enzyme in a non-modified (and non-immobilized) state, so as to later measure improvements (or otherwise) against a reference. There is also an argument here for some standardization (e.g., pH, T, substrate concentration), although since each reaction will operate with a different substrate, the most important aspect of testing the performance of a biocatalyst is to ensure that, initially, what is measured is the intrinsic kinetics, and stability can then follow from this. For example, if the measured activity is not intrinsic, then stability effects may be masked (as can occur with an immobilized enzyme where the observed reaction rate can be controlled by diffusional limitations). Based on intrinsic kinetics, an accurate picture of the enzyme performance can be built. Alteration through protein engineering, immobilization, or altered conditions (to those closer to industry) can then always be compared to these intrinsic data. Historically, several methods have been proposed to collect kinetic data from progress time-curves, even for unstable enzymes [40,41,42]. In fact, as long ago as 1965, Selwyn proposed a simple test to determine the effects of enzyme instability through normalizing time-course data [43]. Of course, the effect of enzyme concentration in itself on stability may also need to be tested, since kinetic experiments are usually performed at low enzyme concentrations. Using these techniques as a basis, more recently in my laboratory, we (and our colleagues) proposed a methodology to ensure that the initial data collected are intrinsic, using a series of progress time-curves under different conditions [44]. This involves, for example, making measurements of progress-time curves under controlled conditions and at decreasing biocatalyst levels to ensure that the rate is not limited by other factors such as mass transfer. The methodology examines each of the factors that affects rate in a systematic way, in order to establish the true intrinsic kinetics of the enzyme. Subsequently, the stability effects, also at higher enzyme loadings, can be examined.

### 6.2. Conditions for Establishing Metrics Closer to Industrial Operation

As emphasized here, since the measured biocatalyst performance is affected by conditions of operation (e.g., pH, temperature, substrate concentration, and enzyme concentration) and process configuration (e.g., substrate feeding and in situ product removal), the development of suitable operating conditions needs to go hand-in-hand with the assessment of biocatalyst performance. Some conditions to evaluate are perhaps obvious, from pH and temperature to varying concentrations of substrate(s) and product(s). However, other conditions are far from obvious. For example, in many items of process equipment, enzymes are exposed to high shear conditions. While it has long been known that shear in itself appears not to damage proteins [45,46], secondary effects can be observed. Likewise, reactions involving multiple phases need particularly careful treatment. In particular, gas-liquid interfaces (present, for example, when delivering oxygen from air in a bubble column or sparged into a stirred tank reactor) are known to lead to enzyme denaturation [47]. Some oxidation reactions require such oxygen supply methods, and reports of denaturation effects have been forthcoming from a number of research groups. More recent reports have examined the denaturation mechanism [48] and the effect of the amount of interface [49,50]. When immobilizing enzymes, such effects will be different, since the enzyme environment is altered; this again emphasizes the importance of determining biocatalyst performance under similar conditions, and with a similar biocatalyst format, to that which will be used in the final process.

Some other, newer cases are where enzyme deactivation requires further study concerning in situ product removal in integrated hybrid equipment. A particularly interesting example is enzymatically-catalyzed, reactive distillation, which is used in enzymatic esterification, where a recent report defined the specific operating conditions needed for future experimentation [51].

### 6.3. Modelling

Engineers, whose primary task is to design and optimize process plants, often make use of mathematical models to describe the phenomena observed in reactors and processes. An important objective in doing so is to predict performance under different conditions, in order to test alternative modes of operation (ahead of experimentation). In fact, long ago, basic models with which to describe enzyme stability were developed (dependent upon the degradation mechanism) [52,53]. These models can form the basis for predictions, although a far wider data set covering different conditions is required. More recently, several attempts have also been made to use such models as the basis of accelerated biocatalyst stability testing using a temperature ramp, capitalizing upon the characteristic of enzymes to rapidly lose stability as temperature is elevated [29,54,55]. Protein engineering has gained enormously from the implementation of high-throughput methods and tools, many of which are automated; the majority are focused on activity measurements. The need now is to complement this with more detailed kinetic characterization, and ultimately, with stability measurements.

## 7. Concluding Remarks

The need for standardization, both in terms of the measures used to assess biocatalyst performance and the way in which the measurements are made, and most importantly, also in terms of the conditions to which a biocatalyst is exposed, will become essential as more comparisons and benchmarks are made between enzyme variants, alternative catalysts, and options for alternative routes to products. Indeed, in the field of enzyme immobilization in particular, there is a need for standardized reporting of biocatalyst performance data, so as to compare one system with another. Such data also need to be more comprehensive and include information such as protein loading, diffusion, observed and intrinsic activity, as well as data on particle size, pore size, and compressibility.

**Figure 2:**
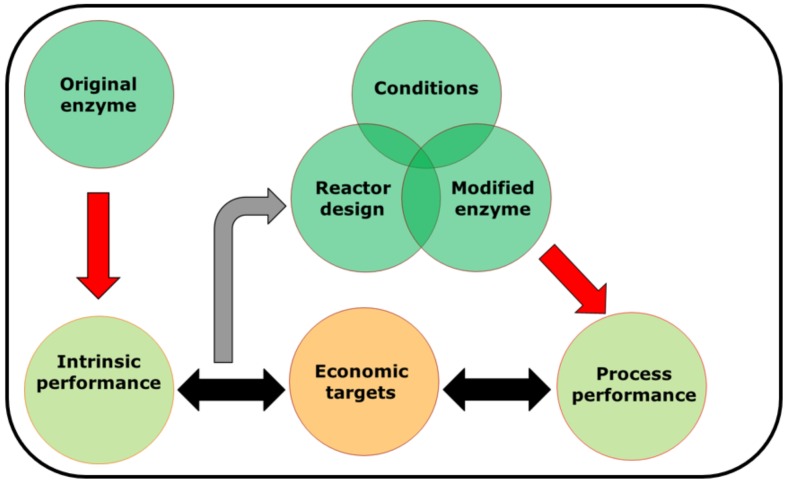
Schematic representation of strategy for repeated measurement of biocatalyst performance (biocatalyst yield, productivity, achievable product concentration).

At first sight, measuring catalyst efficiency seems very straightforward. Conventionally, biochemists have used the catalytic efficiency, and process chemists, the turnover number. However, as highlighted in this brief article, such metrics, while useful, do not tell the whole story. Indeed, it is important to establish the TTN of an enzyme in concert with its productivity and achievable product concentration. In fact, it appears that the greatest use can be made of such data when all three performance metrics are reported together (much as thermodynamic ‘constants’ are reported, for example, under given conditions). It seems likely that a methodology should start with measuring the intrinsic biocatalyst performance, and should be repeated as modifications to the enzyme (through protein engineering or immobilization), reactor configuration, or operating mode are implemented. The ultimate objective is to find an economically-relevant amount of enzyme which is capable of producing the product to the desired concentration in the required time. However, this can be done rapidly (high activity, low operational stability) or slowly (low activity, high operational stability). Other considerations will determine which of these options is best. A schematic representation of a potential systematic methodology is shown in Figure 2. In order to use such data for design and development, it is essential that measurements of performance are made and repeated throughout the improvement cycle, both to set targets for improvement (alongside the required economic performance), but also to measure progress towards this objective. Such a tool has great value, not only for laboratory-scale synthesis, but also in industrial scale development.

## Figures and Tables

**Table 1:** Various reported methods of measuring biocatalyst performance. All methods are dependent upon the conditions used, and therefore, should not be quoted in isolation. * Measured over the lifetime of the biocatalyst. ** Achievable product concentration and productivity.

Metric	Definition	Advantages	Main Limitations
k_cat_/K_M_	mol P/(mol Biocat.t.K_M_)	Biochemists standard measurement	Disregards stability
TON	mol P/mol Biocat	Often used to compare with other enzymes	Disregards rate
TOF	mol P/mol Biocat.t	Good for stable enzymes	Disregards stability
TTN *	mol P/mol Biocat	Good for laboratory use with pure enzymes	Disregards enzyme purity
BY *	mass P/mass B	Good for economic assessment	Disregards other metrics **
